# Supplementary material for: Allosteric regulation and crystallographic fragment screening of SARS-CoV-2 NSP15 endoribonuclease
Source: Nucleic Acids Res. 2023 Apr 28;51(10):5255–70. doi: 10.1093/nar/gkad314 (PMC10250223; doi:10.1093/nar/gkad314)
Supplement: gkad314_Supplemental_Files [file gkad314_supplemental_files.zip › Supplementary datasets.zip!/139/Refine_1/01-BUSTER/Cycle-4/shell.01/R_Rfree.html]

# Rxpct factors in resolution bins at cycle 0


|  |  |  |  |  |  |  |  |  |
| --- | --- | --- | --- | --- | --- | --- | --- | --- |
|  | **<d> (Å)**  Rxpct\_Work | Rxpct\_Free | Rxpct\_All | |  |  |  |  |
|  | 13.2733 | 0.3207 | 0.4720 | 0.3271 |  |
|  | 7.6634 | 0.2594 | 0.2585 | 0.2593 |  |
|  | 5.9360 | 0.2794 | 0.2915 | 0.2799 |  |
|  | 5.0168 | 0.2244 | 0.2371 | 0.2252 |  |
|  | 4.4244 | 0.1960 | 0.2204 | 0.1973 |  |
|  | 4.0021 | 0.1920 | 0.1978 | 0.1923 |  |
|  | 3.6814 | 0.2005 | 0.2419 | 0.2024 |  |
|  | 3.4272 | 0.2049 | 0.2139 | 0.2054 |  |
|  | 3.2193 | 0.1978 | 0.2280 | 0.1994 |  |
|  | 3.0451 | 0.1984 | 0.2101 | 0.1990 |  |
|  | 2.8965 | 0.2021 | 0.2211 | 0.2030 |  |
|  | 2.7677 | 0.1992 | 0.2142 | 0.1999 |  |
|  | 2.6547 | 0.2062 | 0.2203 | 0.2070 |  |
|  | 2.5545 | 0.1993 | 0.2245 | 0.2007 |  |
|  | 2.4648 | 0.1963 | 0.2201 | 0.1975 |  |
|  | 2.3840 | 0.1983 | 0.2294 | 0.1997 |  |
|  | 2.3106 | 0.1933 | 0.1837 | 0.1929 |  |
|  | 2.2436 | 0.1948 | 0.2035 | 0.1953 |  |
|  | 2.1821 | 0.1886 | 0.1796 | 0.1882 |  |
|  | 2.1254 | 0.1947 | 0.1918 | 0.1946 |  |
|  | **Overall** | 0.2136 | 0.2339 | 0.2146 |  |

# Rxpct factors in resolution bins at cycle 1


|  |  |  |  |  |  |  |  |  |
| --- | --- | --- | --- | --- | --- | --- | --- | --- |
|  | **<d> (Å)**  Rxpct\_Work | Rxpct\_Free | Rxpct\_All | |  |  |  |  |
|  | 13.2733 | 0.3212 | 0.4734 | 0.3276 |  |
|  | 7.6634 | 0.2598 | 0.2580 | 0.2597 |  |
|  | 5.9360 | 0.2796 | 0.2909 | 0.2800 |  |
|  | 5.0168 | 0.2243 | 0.2378 | 0.2251 |  |
|  | 4.4244 | 0.1959 | 0.2211 | 0.1972 |  |
|  | 4.0021 | 0.1919 | 0.1974 | 0.1922 |  |
|  | 3.6814 | 0.2001 | 0.2420 | 0.2021 |  |
|  | 3.4272 | 0.2044 | 0.2138 | 0.2049 |  |
|  | 3.2193 | 0.1971 | 0.2277 | 0.1987 |  |
|  | 3.0451 | 0.1977 | 0.2099 | 0.1982 |  |
|  | 2.8965 | 0.2013 | 0.2211 | 0.2023 |  |
|  | 2.7677 | 0.1985 | 0.2142 | 0.1992 |  |
|  | 2.6547 | 0.2055 | 0.2204 | 0.2064 |  |
|  | 2.5545 | 0.1987 | 0.2240 | 0.2002 |  |
|  | 2.4648 | 0.1959 | 0.2201 | 0.1970 |  |
|  | 2.3840 | 0.1980 | 0.2295 | 0.1994 |  |
|  | 2.3106 | 0.1932 | 0.1840 | 0.1928 |  |
|  | 2.2436 | 0.1947 | 0.2037 | 0.1951 |  |
|  | 2.1821 | 0.1886 | 0.1797 | 0.1882 |  |
|  | 2.1254 | 0.1948 | 0.1922 | 0.1947 |  |
|  | **Overall** | 0.2134 | 0.2340 | 0.2144 |  |

# Rxpct factors in resolution bins at cycle 2


|  |  |  |  |  |  |  |  |  |
| --- | --- | --- | --- | --- | --- | --- | --- | --- |
|  | **<d> (Å)**  Rxpct\_Work | Rxpct\_Free | Rxpct\_All | |  |  |  |  |
|  | 13.2733 | 0.3213 | 0.4739 | 0.3278 |  |
|  | 7.6634 | 0.2598 | 0.2579 | 0.2596 |  |
|  | 5.9360 | 0.2793 | 0.2906 | 0.2797 |  |
|  | 5.0168 | 0.2240 | 0.2379 | 0.2249 |  |
|  | 4.4244 | 0.1958 | 0.2213 | 0.1971 |  |
|  | 4.0021 | 0.1917 | 0.1971 | 0.1920 |  |
|  | 3.6814 | 0.1996 | 0.2420 | 0.2016 |  |
|  | 3.4272 | 0.2037 | 0.2134 | 0.2043 |  |
|  | 3.2193 | 0.1963 | 0.2273 | 0.1980 |  |
|  | 3.0451 | 0.1969 | 0.2097 | 0.1975 |  |
|  | 2.8965 | 0.2007 | 0.2211 | 0.2016 |  |
|  | 2.7677 | 0.1978 | 0.2145 | 0.1986 |  |
|  | 2.6547 | 0.2049 | 0.2205 | 0.2059 |  |
|  | 2.5545 | 0.1983 | 0.2238 | 0.1998 |  |
|  | 2.4648 | 0.1955 | 0.2200 | 0.1967 |  |
|  | 2.3840 | 0.1978 | 0.2301 | 0.1993 |  |
|  | 2.3106 | 0.1932 | 0.1841 | 0.1928 |  |
|  | 2.2436 | 0.1946 | 0.2037 | 0.1951 |  |
|  | 2.1821 | 0.1887 | 0.1798 | 0.1883 |  |
|  | 2.1254 | 0.1950 | 0.1925 | 0.1949 |  |
|  | **Overall** | 0.2130 | 0.2339 | 0.2141 |  |

# Rxpct factors in resolution bins at cycle 3


|  |  |  |  |  |  |  |  |  |
| --- | --- | --- | --- | --- | --- | --- | --- | --- |
|  | **<d> (Å)**  Rxpct\_Work | Rxpct\_Free | Rxpct\_All | |  |  |  |  |
|  | 13.2733 | 0.3161 | 0.4786 | 0.3230 |  |
|  | 7.6634 | 0.2545 | 0.2609 | 0.2549 |  |
|  | 5.9360 | 0.2668 | 0.2722 | 0.2670 |  |
|  | 5.0168 | 0.2128 | 0.2206 | 0.2133 |  |
|  | 4.4244 | 0.1845 | 0.2175 | 0.1863 |  |
|  | 4.0021 | 0.1811 | 0.1824 | 0.1811 |  |
|  | 3.6814 | 0.1872 | 0.2318 | 0.1893 |  |
|  | 3.4272 | 0.1878 | 0.2019 | 0.1886 |  |
|  | 3.2193 | 0.1808 | 0.2162 | 0.1828 |  |
|  | 3.0451 | 0.1802 | 0.2068 | 0.1814 |  |
|  | 2.8965 | 0.1841 | 0.2136 | 0.1855 |  |
|  | 2.7677 | 0.1826 | 0.2050 | 0.1836 |  |
|  | 2.6547 | 0.1904 | 0.2174 | 0.1920 |  |
|  | 2.5545 | 0.1903 | 0.2113 | 0.1915 |  |
|  | 2.4648 | 0.1875 | 0.2123 | 0.1886 |  |
|  | 2.3840 | 0.1940 | 0.2285 | 0.1956 |  |
|  | 2.3106 | 0.1918 | 0.1843 | 0.1915 |  |
|  | 2.2436 | 0.1958 | 0.2112 | 0.1966 |  |
|  | 2.1821 | 0.1906 | 0.1879 | 0.1905 |  |
|  | 2.1254 | 0.2009 | 0.2005 | 0.2008 |  |
|  | **Overall** | 0.2024 | 0.2266 | 0.2036 |  |
